# Neuropsychiatric and neural correlates of subjective cognitive complaint

**DOI:** 10.1007/s40520-026-03378-4

**Published:** 2026-03-23

**Authors:** Natascia De Lucia, Tony Thayanandan, Sara Palomba, Klara Komici, Nelson Mauro Maldonato, Giuseppe Rengo, Grazia Daniela Femminella

**Affiliations:** 1 Department of Neurosciences, Reproductive and Odontostomatological Sciences, Federico II University, via S. Pansini 5, Naples, 80131 Italy; 2 Department of Psychiatry, University of Oxford, Oxford, UK; 3 Department of Medicine and Health Sciences, University of Molise, F. De Sanctis snc, Campobasso, 86100 Italy; 4 Department of Translational Medical Sciences, Federico II University, S. Pansini 5, Naples, 80131 Italy; 5 Istituti Clinici ScientificiMaugeri IRCCS-Scientific, Institute of Telese Terme, Telese Terme, BN, Italy; 6 Department of Brain Sciences, Imperial College London, South Kensington Campus SW7 2AZ, London, UK

**Keywords:** Subjective cognitive complaint, Mild cognitive impairment, Brain amyloid, Depression, Anxiety

## Abstract

**Background:**

Subjective cognitive complaint (SCC) has been reported in normal elderly (NE) and Mild Cognitive Impairment (MCI).

**Aims:**

We investigated the neuropsychiatric predictors of SCC in NE and MCI, and the biomarkers abnormalities, and neural correlates of SCC in MCI.

**Methods:**

Clinical, cognitive and imaging data of 233 MCI and 419 NE were obtained from the Alzheimer’s Disease Neuroimaging Initiative 3 (ADNI-3) database. SCC was assessed by the Cognitive Change Index (CCI) and Everyday Cognition (ECog). Neuropsychiatric symptoms were evaluated through the Neuropsychiatric Inventory (NPI). Brain amyloid and tau status were obtained from [18 F]Florbetapir-PET and [18 F]Flortaucipir-PET SUVR in predefined target regions, and brain and grey matter volumes from structural MRI.

**Results:**

SCC was significantly correlated with depression, anxiety, apathy, irritability, and sleep disorders, in MCI. Linear regression showed that depression and anxiety were significantly associated to SCC index, in MCI. SCC was neither significantly different in amyloid positive vs. negative, nor in tau positive vs. negative MCI. MCI with SCC showed significantly lower Braak 3–4 region volume and reduced amygdala volume, compared to MCI without SCC. MCI with SCC and NPI showed lower posterior cingulate cortex volume compared to MCI without SCC or NPI, whereas MCI with SCC but without NPI had lower anterior cingulate cortex volume, compared to MCI without SCC or NPI.

**Conclusions:**

These findings recommend the crucial role of psychological therapies focused on anxiety and depression, to prevent the worsening of the subjective cognitive complaint that represent a strong factor of conversion to objective cognitive disorders.

## Introduction

Subjective cognitive complaint (SCC) refers to self-perception of cognitive change (or sometimes a close informant’s perception) in everyday functioning, very often of memory, without objective cognitive evidence of impairment [1, 2]. SCC has been often observed in older adults (OA) and in individuals suffering from mild cognitive impairment (MCI). In OA, SCC appears to be correlated with depressive affect, anxiety, hypochondriasis, and post-traumatic stress disorder [3, 4]. The subjective experience of cognitive decline has also been associated with specific personality traits in elders, such as neuroticism, low self-esteem, openness and conscientiousness, self-discipline and self-consciousness [5]. A recent study [6] showed that older age, sex, thyroid diseases, anxiety symptoms, lack of physical exercises and living alone, may represent crucial risk factors for SCC in normal elders.

In MCI, SCC has been considered as a crucial criterion for the diagnostic framework of MCI [7, 8]. According to some authors [9] SCC represents a feature of neurobiological changing that predicted the conversion from normal cognition to MCI, and the progressing to dementia [10]. Moreover, SCC in MCI appears to be related to neuropsychiatric symptoms, such as depressive affect, and neuroticism personality trait [9]. In a recent review, some authors [11] observed that when SCC was associated with anxiety or worry then the risk of progression to MCI or dementia increased, whereas higher levels of depressive symptoms were not at a greater risk of progression. Some authors [12] observed that the tendency to maintain positive relations with others and efforts to minimize interpersonal conflicts (agreeableness trait) were negatively associated to SCC. Increased amyloid deposition in brain measured by positron emission tomography (PET) [13–15] more severe brain atrophy measured by magnetic resonance imaging (MRI) were also found in SCC individuals [16].

However, studies exploring the self-perceived cognitive changes in OA and MCI only focused on the clinical features that facilitate the progression from SCC to cognitive decline, whereas no studies investigated the possible relationships between SCC, neuropsychiatric symptoms, and brain morphological features. In the present study, we aimed to investigate the neuropsychiatric predictors of SCC, and the morphological changes in brain volume and grey matter volume associated with the self-perceived cognitive changes in individuals with MCI, and in a sample of OA. This morphometric approach could allow to comprehend the neural bases of SCC, possibly to identify the targets of effective therapies for this disabling clinical condition. Moreover, we aimed to explore the biomarker status of this MCI cohort by the assessment of brain amyloid and tau deposition through PET imaging to evaluate whether increased SCC was associated with biomarkers abnormalities in MCI.

## Methods

### Participants

We obtained data from the third study phases of the Alzheimer’s Disease Neuroimaging Initiative (ADNI-3) database (see adni.loni.usc.edu for more information). ADNI is an ongoing international longitudinal study aimed to identify the markers of early AD and to monitor the AD progression. To qualify for the present study participants had to meet the inclusion criteria for the clinical diagnosis of MCI according to the ADNI guidelines (ADNI-3 Protocol, 2016). Data from a total of 233 (121 female) MCI participants were available. We also included data from 419 (223 female) OA.

### Subjective cognitive complaint assessment

To assess the subjective changes in cognitive function all the participants completed the Cognitive Change Index (CCI) [17], and the Everyday Cognition (ECog) measure [18]. The CCI is a 20 items instrument requiring participants and their informants to rate the memory, executive and language status relative to previous five years. Each item is rated on a 5-point scale where higher scores indicate greater perceived decline, and the CCI total score is derived by combining the participant and study partner sets. The ECog is a 41-item informant-based measure of cognitively-relevant everyday abilities, including: memory, language, visuospatial, planning, organization, and divided attention. For each item, informants were required to compare the participant’s current level of everyday functioning with how he or she functioned 10 years earlier. Each item is rated on a 4-point where higher scores indicate substantial perceived decline in that specific daily cognitive function.

### Cognitive and neuropsychiatric assessment

The cognitive assessment had been performed by means of the following tests: Clinical Dementia Rating (CDR) scale to denote the presence and stage the severity of dementia [19]; Mini Mental State Examination (MMSE) [20], and Montreal Cognitive Assessment (MOCA) [21] for the general cognitive functioning; word recall, word delayed recall and recognition tests of the Alzheimer ‘s disease Assessment Scale - Cognition (ADAS-Cog) [22] and immediate recall, delayed recall and recognition of the Rey auditory verbal learning test [23] for memory abilities; ADAS-Cog naming test (Rosen et al., 1984), and Category fluency animals test [24] for language abilities; ADAS-Cog copying designs [22], and Clock drawing test [25] for visuo-spatial abilities; ADAS-Cog number cancellation^,^ [22] and Trail making test [26] for executive abilities. The neuropsychiatric assessment had been performed by means of the Neuropsychiatric Inventory (NPI) [27], an informant-report structured interview assessing frequency (on a 5-point scale) and severity (on a 3-point scale) of: delusions, hallucinations, agitation/aggression, depression, anxiety, euphoria, apathy, disinhibition, irritability, aberrant motor behaviour, sleep, eating. The total-domain score was then obtained by multiplying frequency and severity scores, whereas the total NPI score was calculated by adding the individual total-domain scores (score range: 0-144).

### Neuroimaging data

The [18 F]Florbetapir-PET images had been acquired using Siemens, GE and Philips PET scanners with a standard dynamic 50–70 min protocol following the intravenous injection of 370 ± 37 MBq of [18 F]-AV45. The summary SUVR value normalized by the whole cerebellum with a cutoff of 1.1 was used to determine Aβ-positivity/negativity. The normalized Flortaucipir SUVRs used in this study were generated by dividing the SUVR in the region of interest by the inferior cerebellar GM SUVR. To determine tau positivity/negativity, the inferior temporal cortex (ITC) was selected as target ROI. The cut-off for Flortaucipir positivity was set as ITC tau PET > 2.122 SUVR. Regional SUVR data extraction has been performed as previously described [28]. Volumetric regional data were collected from structural brain MRI scans, segmented with Freesurfer, as previously described [28].

### Statistical analyses

We computed number of participants showing neuropsychiatric symptoms. To compare demographic (age and education level) and clinical (global CDR scores) data between OA and MCI groups we performed independent samples t-tests and Mann-Whitney U test, respectively. Then we performed a multivariate analysis of variance (MANOVA) to compare cognitive, neuropsychiatric, and SCC test scores, in OA and MCI individuals. Then, to identify the variables associated with SCC we ran a Spearman’s correlation analysis between neuropsychiatric test scores and SCC index, obtained from the mean of the Z-scores for CCI and Ecog measures, in OA and MCI individuals. Then, to investigate which specific NPI domain scores had independent associations with SCC we performed multiple linear regression models, with enter method. To run this analysis, we considered the SCC index as dependent variable, whereas the NPI domain scores that showed significant bivariate correlations with the SCC index were simultaneously entered as independent variables. The regression models were adjusted for sex, age, and education level as potential confounding variables. Last, we stratified our MCI population according to presence of neuropsychiatric symptoms and subjective cognitive complaint into four groups. In particular, the presence or absence of neuropsychiatric symptoms was determined according to established cut-off values of the NPI scale (i.e., NPI total score > 0) [27], whereas presence of SCC was identified by using a cut-off derived from the control group mean minus 1.5 standard deviations, according to conventional neuropsychological criteria [29]. Prevalence of amyloid positive subjects in the four groups was assessed through χ2 test. Regional brain Flortaucipir SUVR and regional volumes were compared in the four groups with ANOVA with Bonferroni *post-hoc* correction.

## Results

In OA, 113/419 (26.9%) participants showed neuropsychiatric symptoms, with: 59/419 (14%) showing sleep and nighttime behaviour disturbances, 42/419 (10%) depression/dysphoria, 33/419 (7.8%) irritability/lability, 17/419 (4%) anxiety, 16/419 (3.8%) agitation/aggression, 15/419 (3.5%) apathy/indifference, 12/419 (2.8%) appetite/eating disorders, and 5/419 (1.1%) disinhibition. In MCI, 142/233 (61%) individuals showed neuropsychiatric symptoms, with: 61/233 (26.1%) presenting depression/dysphoria, 54/233 (23.1%) sleep and nighttime behaviour disturbances, 54/233 (23.1%) irritability/lability, 37/233 (15.8%) apathy/indifference, 32/233 (13.7%) agitation/aggression, 30/233 (12.8%) anxiety, 28/233 (12%) disinhibition, 21/233 (9%) appetite/eating disorders, 8/233 (3.4%) aberrant motor behaviour, 4/233 (1.7%) euphoria/elation, 1/233 (0.4%) delusions, 1/233 (0.4%) hallucinations. The proportion of participants showing neuropsychiatric symptoms differed significantly in the two groups, χ^2^(1,652) = 72.7, *p*<.001, φ = 0.33.

The comparison between MCI and OA showed that the two groups did not significantly different in age, t(650) = − 1.877, *p*=.06, Cohen’s d = 0.15, or education level t(650) = 3.173, *p*=.15, Cohen’s d = 0.25. A Mann–Whitney U test showed that global CDR scores were higher in the MCI group than in the OA group, U = 47,443, Z = − 3.12, *p*<.001, *r* = .13. Results from MANOVA (see Table 1) showed a significant effect of the diagnosis (Wilks’ lambda = 0.626; *p*<.001; η^2^_p_ = 0.37). MCI showed significantly poorer performance on clinical and cognitive test scores compared with OA (all the comparisons: *p*<.005, after Bonferroni-corrected *post-hoc*). On neuropsychiatric assessment, MCI showed significantly higher scores on agitation/aggression, depression/dysphoria, anxiety, euphoria/elation, apathy/indifference, disinhibition, irritability/lability, aberrant motor behaviour, sleep and nighttime behaviour disorders, and appetite/eating behaviour, compared with OA, whereas no significant differences were observed on delusions, and hallucinations. Moreover, MCI achieved significantly higher scores on CCI and ECog tests compared with OA.

**Table 1:** Mean and standard deviation (SD) on demographic, clinical, cognitive and neuropsychiatric data in older adults (OA) and mild cognitive impairment (MCI) individuals

		OA (*N* = 419)		MCI (*N* = 233)	
	Range score	Mean	SD	Mean	SD
***Demographic and clinical data***					
Age	-	77.1	7.2	78.4	8.2
Education level	-	16.8	2.3	16.2	2.6
Clinical dementia rating scale	0–3	0.03	0.22	1.4*	1.2
***Cognitive tests***					
Mini Mental State Examination	0–30	29.0	1.1	27.9*	2.0
Montreal Cognitive Assessment	0–30	26.2	2.6	23.0*	3.3
ADAS-cog word recall	0–10	2.7	1.3	4.1*	1.4
ADAS-cog word delayed recall	0–10	2.5	1.8	4.9*	2.4
ADAS-cog word recognition	0–12	3.5	2.4	1.8*	1.8
RAVLT - immediate recall		46.6	10.5	36.0*	10.4
RAVLT- learning		6.0	2.4	4.2*	2.6
RAVLT - delayed recall		4.5	3.5	3.5*	3.2
ADAS-cog naming	0–5	0.1	0.4	0.0*	0.2
Category fluency animals		21.7	5.4	18.3*	5.1
ADAS-cog copying geometric designs	0–5	0.5	0.6	0.4*	0.5
Clock drawing test		4.7	0.5	4.3*	0.8
ADAS-cog number cancellation	-	0.7	0.9	0.3*	0.6
Trail making test – part A (time)		30.2	8.8	37.9*	15.2
Trail making test – part B (time)		72.5	33.9	104.1*	59.1
***Neuropsychiatric assessment***					
Delusions	0–12	0.0	0.0	0.1	0.1
Hallucinations	0–12	0.0	0.0	0.0	0.0
Agitation/aggression	0–12	0.1	0.1	0.3*	1.1
Depression/dysphoria	0–12	0.2	0.8	0.5*	1.3
Anxiety	0–12	0.1	0.4	0.3*	1.2
Euphoria/elation	0–12	0.0	0.0	0.0*	0.2
Apathy/indifference	0–12	0.1	0.7	0.5*	1.4
Disinhibition	0–12	0.0	0.4	0.2*	0.9
Irritability/lability	0–12	0.1	0.8	0.7*	1.7
Aberrant motor behaviour	0–12	0.0	0.0	0.0*	0.6
Sleep and nighttime behaviour disorders	0–12	0.4	1.1	0.7*	1.9
Appetite/eating behaviour	0–12	0.1	0.8	0.3*	1.4
***Subjective cognitive complaint assessment***					
CCI	1-100	28.1	6.9	48.8*	17.8
ECog	1-156	72.5	42.4	79.8*	45.6
***Biomarkers status***					
Amyloid PET positive, n (%)	-	75/262 (28.6%)	-	60/137 (43.8%)*	-
Tau PET positive, n (%)	-	13/419 (3.1%)	-	43/233 (18.5%)*	-

*significant different from OA at *p*<.005

The correlation analysis, in MCI, revealed that increased SCC was significantly and positively associated with depression/dysphoria, anxiety, apathy/indifference, irritability/lability, and sleep disorders. In OA, no significant correlations were observed. Spearman’s correlations, as estimates of effect sizes, are reported in Table 2.

**Table 2:** Spearman’s correlation coefficients between scores on individual domains of the Neuropsychiatric Inventory and the SCC index, in MCI and OA individuals

	MCI (*N* = 233)	OA (*N* = 419)
Neuropsychiatric Inventory		
Delusions	− 0.007	-
Hallucinations	0.002	-
Agitation/aggression	0.016	0.032
Depression/dysphoria	0.122^*^	0.067
Anxiety	0.135^*^	0.033
Euphoria/elation	− 0.047	0.039
Apathy/indifference	0.163^*^	0.015
Disinhibition	− 0.055	0.047
Irritability/lability	0.143^*^	0.000
Aberrant motor behaviour	0.041	0.035
Sleep and nighttime behaviour disorders	0.158^*^	0.045
Appetite/eating behaviour	0.044	0.007

*significant at *p*<.05

Results from the multiple linear regression model, exploring the independent contribution of depression/dysphoria, anxiety, apathy/indifference, irritability and sleep disorders in predicting the SCC in MCI showed that the overall model was statistically significant, F(8,224) = 4.430, *p*<.001, R^2^ = 0.052; depression/dysphoria and anxiety symptoms were significantly associated to SCC index. Regression coefficients, standard errors, and *p*-values for all included NPI domains are reported in Table 3. Regression analyses were performed only in the patient group, as no significant bivariate associations were found in the control group.

**Table 3:** Summary of the multiple linear regression model for SCC index in individuals with mild cognitive impairments (MCI)

Predictor	SE	β	t	*p*
Sex	0.058	0.052	1.293	0.19
Age	0.004	0.070	1.772	0.07
Education	0.011	0.028	0.707	0.48
NPI – Depression/dysphoria	0.129	0.138	2.257	0.002
NPI - Anxiety	0.034	0.128	3.198	0.001
NPI – Apathy/indifference	0.030	0.023	0.522	0.60
NPI – Irritability/lability	0.025	0.059	1.358	0.17
NPI - Sleep and nighttime behaviour disorders	0.018	0.070	1.732	0.08

NPI: neuropsychiatric inventory; SCC: subjective cognitive complaint; β: standardized coefficient; SE: standard error

**Fig. 1:**
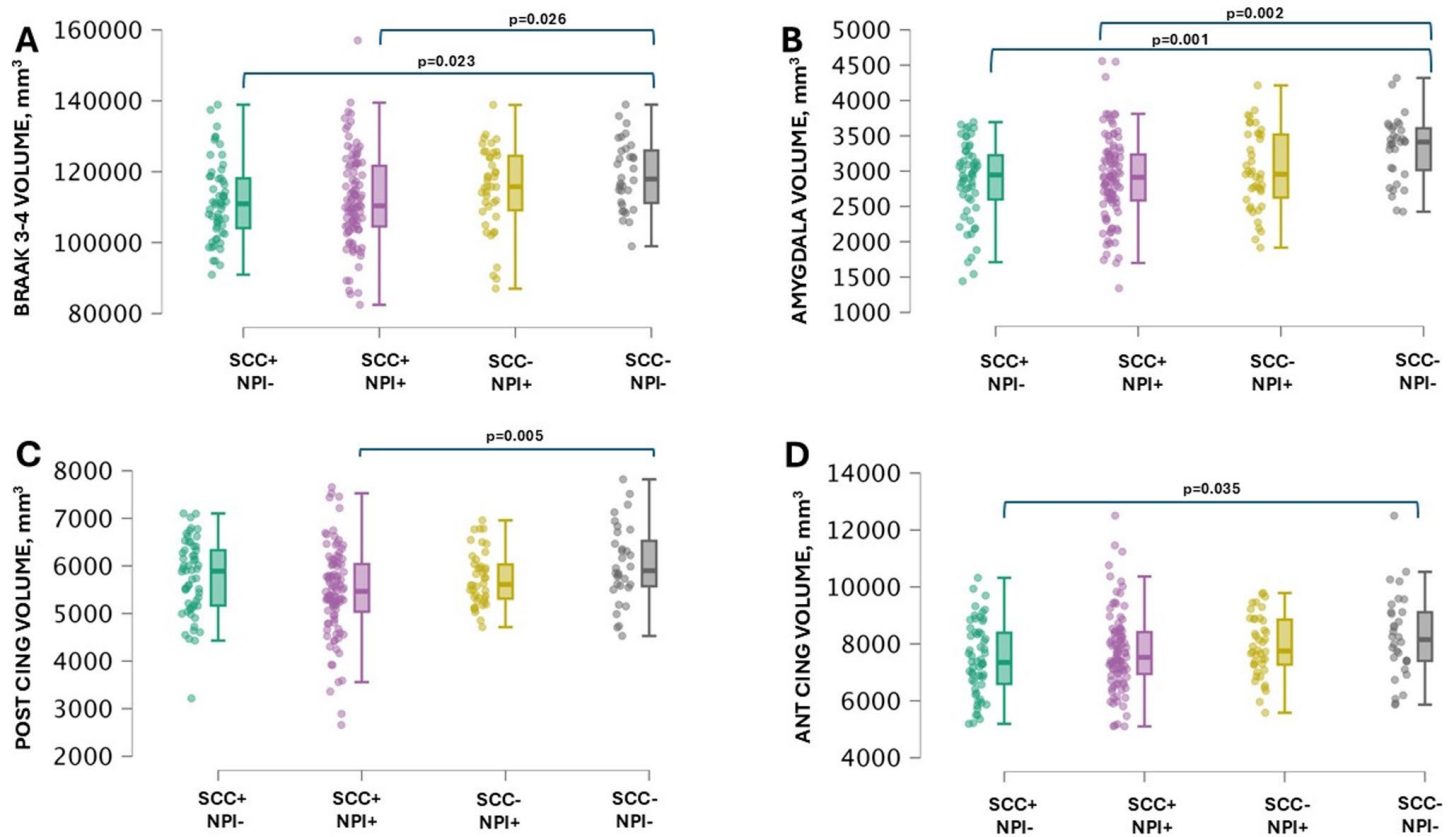
Volumetric regional data in MCI subgroups. Regional volumes from structural brain MRI scans, segmented with Freesurfer, for Braak 3–4 region (**A**), amygdala (**B**), posterior cingulate (**C**) and anterior cingulate (**D**) cortex were compared among the four MCI subgroups. Volumes are expressed in mm3, *p*-values are corrected with Bonferroni correction

Finally, in MCI 43.8% of subjects had a positive amyloid PET scan, and 18.5% had positive Tau PET. The SCC index was neither significantly different in amyloid positive vs. negative (47.8 ± 17.9 vs. 53.2 ± 18.6), nor in tau positive vs. negative (45.2 ± 16.5 vs. 35.2 ± 15.5) MCI subjects. When stratifying MCI subjects according to presence or absence of SCC and presence or absence of NPI, 87 participants were included within the SCC+/NPI- group, 108 participants within the SCC+/NPI+ group, 147 participants within SCC-/NPI + and 308 participants within the SCC-/NPI- group. The comparisons of regional Flortaucipir SUVR among the four groups did not show significant differences in any of the predefined regions. When looking at differences in brain volumes, both SCC+/NPI + and SCC+/NPI- groups showed significantly lower Braak 3–4 region volume compared to SCC-/NPI- group (*p* = .014) (Fig. 1A), as well as significantly reduced amygdala volume (*p* = .01) (Fig. 1B). Moreover, SCC+/NPI+ group showed lower posterior cingulate cortex volume compared to SCC-/NPI- (*p* = .005) (Fig. 1C), and SCC+/NPI- had lower anterior cingulate cortex volume compared to SCC-/NPI- (*p* = .040) (Fig. 1D).

## Discussion

Interest in SCC revamped in the recent years. However, most of the studies focused on the role of the subjective complain in the conversion process to cognitive decline. It has been reported that individuals with SCC and higher level of anxiety have an increased risk of cognitive progression to either MCI or dementia, compared with individuals without SCC [11]. In the present study, instead, we were interested in expanding the spectrum of the clinical features associated with SCC by exploring the neuropsychiatric factors and brain morphological features associated with SCC, in OA and individuals suffering with MCI. Results from MANOVA revealed that MCI individuals, compared with OA, showed poorer performance on tests assessing memory, language, visuo-spatial, and executive abilities, and more frequent and severe neuropsychiatric symptoms. These results were consistent with previous findings [30]. Interestingly, we also observed that MCI individuals achieved significantly higher scores on both CCI and ECog tests, compared with OA. The subjective cognitive complaint appeared to be more severe in MCI, compared with OA, thus confirming previous findings [12]. When we explored for possible correlations between SCC and specific neuropsychiatric symptoms, we observed a strong association of the subjective experience of cognitive changing with more severe level of depression, anxiety, apathy, irritability and sleep disorders, in MCI individuals only. However, when we statistically weighted out the individual contribution of these neuropsychiatric symptoms on the SCC index, we observed that the only significant variables associated with the subjective cognitive decline were the depression affect and anxiety symptom. Thus, higher level of anxiety and more severe depression may predict a higher tendency to subjectively perceive own cognitive functioning as much worse, with respect to the past. These results could also suggest the idea that in MCI individuals an excessive focalization of the attention principally on own emotional experiences with a more marked tendency to ruminate on own past experiences and overthink, with excessive worry and apprehension, on every day and future situations, could contribute to a distorted perception and catastrophic interpretation of own cognitive functioning, stimulating the belief for an actual and severe cognitive difficulty. This would be consistent with previous findings suggesting that the affective disorders, particularly depression, amplify the negative subjective evaluation of cognitive decline [31, 32]. Our findings from brain morphological analysis suggested that SCC are associated with subtle structural brain alterations in individuals with MCI. Although no significant differences in regional PET SUVRs were observed among the four subgroups, both SCC+/NPI + and SCC+/NPI– group exhibited reduced volumes in the Braak stage 3–4 regions and amygdala compared with the SCC–/NPI– group. These results indicate that SCC may reflect early neurodegenerative changes even when molecular markers do not yet differ across groups. Notably, the additional reduction of posterior cingulate cortex volume in the SCC+/NPI+ subgroup may point to a synergistic effect of SCC and NPS on vulnerability of key nodes within the limbic–default mode network. Given the relevance of the posterior cingulate in early Alzheimer’s disease [33], this finding may suggest that the combination of SCC and NPS identifies individuals at heightened risk for progression. Overall, the absence of Florataucipir SUVR differences might be due to the low prevalence of positive tau PET in this population, minimizing differences among subgroups. However, detectable volumetric changes among MCI subgroups suggest that structural MRI may capture early neurodegenerative alterations associated with subjective and neuropsychiatric symptoms in MCI, in critical structures associated with AD pathophysiology. In this cohort, we were not able to show an association of SCC with worse biomarkers profile, as the amyloid and tau status in this MCI group was not associated with differences in SCC index. The longitudinal evaluation of this cohort and others with similar characteristics might help better understand the neurobiological correlates of SCC. Moreover, to contextualize the absence of an association between global SCC index and amyloid/tau status in our cohort, it is important to consider that biomarker relationships in pre-dementia stages may depend on the specific clinical phenotype captured by the instrument. In this regard, Rochetti et al. [34] investigated neuropsychiatric symptom domains using the Mild Behavioral Impairment Checklist (MBI-C) in individuals with SCD and MCI and reported a significant association between the impulse dyscontrol domain (MBI-C domain C) and CSF amyloid-β1–42 concentrations, while no associations were found with tau biomarkers. These findings suggest that symptom-specific neuropsychiatric profiles, particularly impulse dyscontrol, may show closer coupling with amyloid-related pathology than global self-reported complaint severity measures. Differences in measurement strategy, biomarker modality (continuous CSF values versus dichotomized amyloid/tau status), and sample composition may contribute to discrepant findings across studies. Taken together, this supports a non-univocal relationship between subjective complaints and AD biomarkers and highlights the potential utility of stratifying pre-dementia populations by specific neuropsychiatric symptom clusters when probing biomarker associations.

Some limitations have to be considered. We did not perform a qualitative evaluation of the psychopathological symptoms thus further studies could consider to assess the association of SCC with frequency and severity of each clinical sign. Moreover, since our sample size was relatively small, future studies could consider to explore larger sample in order to granter generalizability of findings.

In conclusion, this study provided evidence that anxiety and depression may predict SCC, in individuals suffering with MCI. Conversely, no significant associations were found in older adults. To explore the relationship between emotional features and SCC could contribute to expanding the spectrum of the clinical manifestations of SCC, and help to develop efficacious psychological therapies targeted to prevent the worsening of the subjective decline, that is a crucial factor for the progression of the cognitive decline, and improve the quality of life of MCI individuals.

## Data Availability

Data used in this research is available upon request athttps://adni.loni.usc.edu/.
